# Low Levels of Serum Total Vitamin B12 Are Associated with Worse Metabolic Phenotype in a Large Population of Children, Adolescents and Young Adults, from Underweight to Severe Obesity

**DOI:** 10.3390/ijms242316588

**Published:** 2023-11-22

**Authors:** Alessia Aureli, Rosanna Recupero, Michela Mariani, Melania Manco, Francesco Carlomagno, Sarah Bocchini, Mirella Nicodemo, Maria Rosaria Marchili, Stefano Cianfarani, Marco Cappa, Danilo Fintini

**Affiliations:** 1 Endocrinology and Diabetology Unit, “Bambino Gesù” Children’s Hospital, IRCCS, 00146 Rome, Italy; alessia.aureli@opbg.net (A.A.); michela.mariani@opbg.net (M.M.); sarah.bocchini@opbg.net (S.B.); mirella.nicodemo@opbg.net (M.N.); stefano.cianfarani@uniroma2.it (S.C.); danilo.fintini@opbg.net (D.F.); 2 Pediatric Unit, “Bambino Gesù” Children’s Hospital, IRCCS, 00146 Rome, Italy; rosanna.recupero@opbg.net; 3 Pediatrics Department, University of Rome “Tor Vergata”, 00133 Rome, Italy; 4 Research Area for Foetal Neonatal and Cardiological Sciences, “Bambino Gesù” Children’s Hospital, IRCCS, 00146 Rome, Italy; 5 Department of Experimental Medicine, Sapienza University of Rome, 00161 Rome, Italy; francesco.carlomagno@uniroma1.it; 6 Department of Emergency Admission and General Pediatrics, “Bambino Gesù” Children’s Hospital, IRCCS, 00165 Rome, Italy; mrosaria.marchili@opbg.net; 7 Department of Systems Medicine, University of Rome “Tor Vergata”, 00133 Rome, Italy; 8 Department of Women’s and Children’s Health, Karolinska Institutet, 17177 Stockholm, Sweden; 9 Research Area of Innovative Therapies in Endocrinopathies, “Bambino Gesù” Children’s Hospital, IRCCS, 00146 Rome, Italy; marco.cappa@opbg.net

**Keywords:** vitamin B12, cobalamin, body weight, obesity, underweight, visceral adiposity, lipids, glucose metabolism, insulin resistance, steatosis, liver injury

## Abstract

Vitamin B12 (or cobalamin) is an essential vitamin for DNA synthesis, fatty acid and protein metabolism as well as other metabolic pathways fundamental to the integrity of cells and tissues in humans. It is derived from the diet and mostly stored in the liver. Its deficiency has been associated with metabolic derangements, i.e., obesity, glucose intolerance, increased lipogenesis and metabolic dysfunction-associated steatotic liver disease (MASLD) and steatohepatitis (MASH). However, data with regard to body weight across the whole spectrum (from underweight to severe obesity) in children and young individuals are scarce. The present study aims to describe the association between serum total vitamin B12 and body mass index (BMI) ranging from underweight to severe obesity in a large population of children, adolescents and young adults. This study also investigates associations with visceral adiposity, glucose and lipid metabolism and liver dysfunction. A cross-sectional, single-centre study was conducted at the Paediatrics and Endocrinology units of the ”Bambino Gesù Children Hospital”, a tertiary referral institution for eating disorders. Clinical charts were reviewed and 601 patients aged from 5 to 25 years were enrolled in order to analyse anthropometric, auxological, clinical, biochemical and liver ultrasound data using robust statistical approaches. Analyses were adjusted for potential confounders. A reduction in serum total B12 levels was associated with a linear increase in body weight, as expressed by WHO BMI SDS (r = −0.31, *p* < 0.001, BCa 95% −0.38, −0.24). Lower B12 levels were associated with higher waist circumference but only in pubertal girls (r = −0.33, *p* = 0.008, BCa 95% −0.53, −0.11). Hepatic insulin resistance was higher in males with lower B12 levels (B = −0.003 (−0.007, −0.0001), *p* = 0.039), but not in females, whereas whole-body insulin resistance was unaffected. Serum lipid profiles (total, HDL and LDL cholesterol and triglycerides) were not influenced by serum cobalamin levels. However, lower cobalamin levels were associated with higher grading of ultrasound-scored hepatic steatosis (*p_trend_* = 0.035). Lastly, both AST and ALT showed a significant and direct correlation with total B12 levels in underweight (r = 0.22 and 0.24, *p* = 0.002 and <0.001, respectively) and severely obese subjects (r = 0.24 and 0.32, *p* = 0.002 and <0.001). In conclusion lower vitamin B12 levels are associated with higher body weight, adiposity and with worse metabolic health in a large population of children, adolescents and young adults.

## 1. Introduction

Vitamin B12 (or cobalamin) is an essential vitamin, i.e., derived exclusively from diet. Its principal sources are meat (red meat and poultry, in particular) and animal-derived foods (e.g., milk, cheese and eggs). It can also be found in seafood, such as shellfish and crab [1,2]. The bioavailability of dietary cobalamin is approximately 1.5–2 μg per meal [1,3]. Its absorption occurs in the terminal tract of the small intestine, where it arrives bound to the intrinsic factor (IF) and is absorbed by intestinal microvilli through specific receptors [4]. Circulating cobalamin, together with its main binding protein transcobalamin (TC) forms the holo-transcobalamin (Holo-TC) complex, which is internalised through its receptor expressed on several cell types, including hepatocytes [5,6]. Vitamin B12 is mainly stored in the liver, with a mean content of approximately 2 μg per gram of wet hepatic tissue [7]. After endocytosis, cobalamin is released from the lysosome into the cytosol through the action of ABCD4, belonging to the superfamily D of ATP-binding cassette transporters. These are localised in peroxisomes and lysosomes and are involved in the metabolism of long-chain and very-long-chain fatty acids [8]. In the cytosol, cobalamin is processed to its catalytic forms that are key in regulating gene expression and promoting the survival and integrity of human cells and tissues. Homocysteine (Hcy) and methylmalonic acid (MMA) are two of the most important products of the cobalamin metabolism and are both sensible circulating markers of vitamin B12 deficiency [2,9].

Currently, there is no consensus on the definition of ”vitamin B12 deficiency”, with lower reference values ranging from 120 to 200 pmol/L. Similarly, the upper reference limit fluctuates from 650 to 850 pmo/L [2,10].

Evidence shows that B12 deficiency is associated with increased lipogenesis and reduced lipolysis [11,12,13,14,15,16,17], leading to an atherogenic lipid profile. Animal models demonstrated higher visceral adiposity [11] and worse body composition [18] in offspring born to mothers with B12 deficiency. Likewise, reduced plasma cobalamin has been linked to obesity in children, adults and women in early stages of pregnancy [16,19,20,21,22]. In the latter, increased body fat in offspring has been associated with insulin resistance (IR) and increased risk for cardiovascular disease in adulthood [16,17,23].

Cobalamin deficiency has also been associated with increased serum Hcy, a well-recognized marker of increased cardiovascular risk [24], and a relationship between dietary vitamin B group intake and both cardiovascular mortality and morbidity has been described [25,26,27]. As such, it has been suggested that the beneficial effects of B12 and B9 (folate) supplementation are exerted through decreasing Hcy levels, improving IR and endothelial dysfunction [28,29,30].

Cobalamin deficiency conditions have also been linked to altered one-carbon metabolism and mitochondrial dysfunction, as causes of advanced hepatic fibrosis [9,31]. On the other hand, the association of B12 status with metabolic dysfunction-associated steatotic liver disease (MASLD) and steatohepatitis (MASH) is still debated, with some studies finding lower cobalamin levels in MASLD and MASH subjects [31,32], and others suggesting no difference [33,34]. The association between Hcy and MASH has been somewhat poorly explored, being still a matter of debate [31,35]. Even though the evidence is still controversial, some authors have proposed cobalamin as an independent predictor of MASH histological severity [35] or liver damage in chronic hepatitis [36].

In eating disorders, and particularly in anorexia nervosa (AN), higher plasma cobalamin levels have been described, which are inversely associated with the severity of food restriction, rather than body mass index (BMI) [37]. As such, increased cobalamin levels have been interpreted as early markers of liver injury, due to hepatocyte cytolysis and reduced hepatic clearance of the vitamin [38]. Furthermore, in AN patients with hepatic insufficiency, increased B12 levels predict a higher mortality rate at a 3-month follow-up [39]. Hepatic damage also occurs during refeeding, with hypertransaminasemia inversely proportional to severity of underweight [40].

Considering the evidence of a potential role of vitamin B12 status in the development of metabolic dysfunction in patients suffering from AN, the aim of this study was to explore the relationship between serum B12 levels and metabolic phenotype in a large population of children, adolescents and young adults, ranging from underweight to severe obesity.

## 2. Results

Age, anthropometrics and biochemical parameters of study participants according to weight group are presented in Table 1. Male participants represented the 39.4% of the whole population, and 30.1% of subjects were pre-pubertal.

### 2.1. Body Weight and Visceral Adiposity According to Vitamin B12 Levels

**Figure 1:**
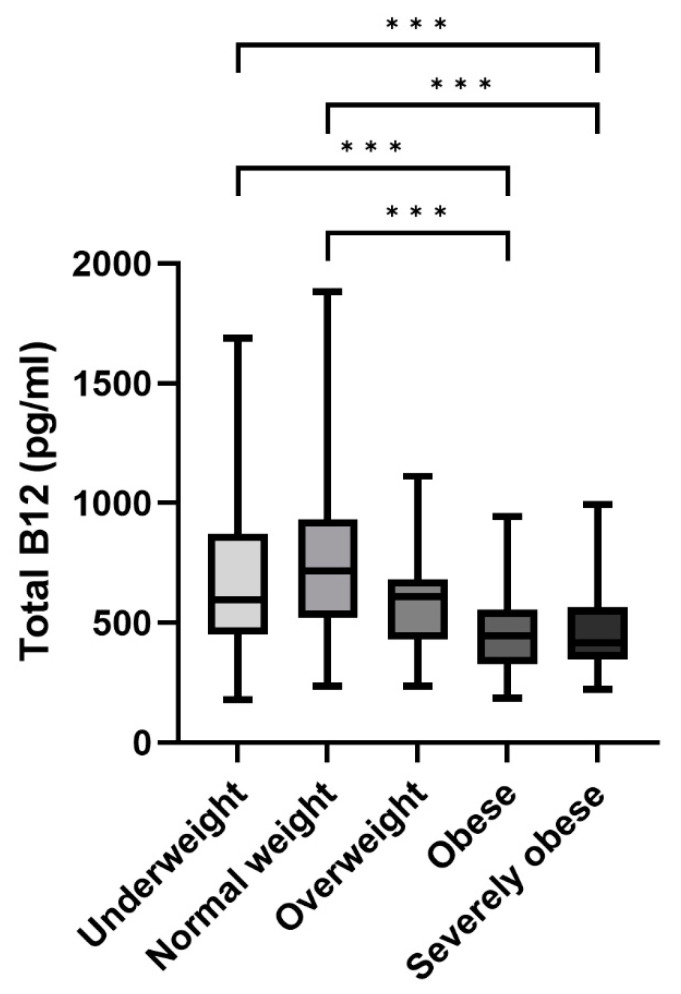
Serum total vitamin B12 levels according to weight status. Note: Box-whisker plots show median (black lines), 25–75% IQR (boxes), and 2.5–97.5th percentiles (whiskers). (*** *p* < 0.001).

Serum total cobalamin levels differed significantly in the population, according to body-weight groups as shown in Figure 1. Specifically, people belonging to the underweight and normal weight groups had significantly higher B12 levels than participants with obesity and severe obesity (*p* < 0.001 for all).

**Figure 2:**
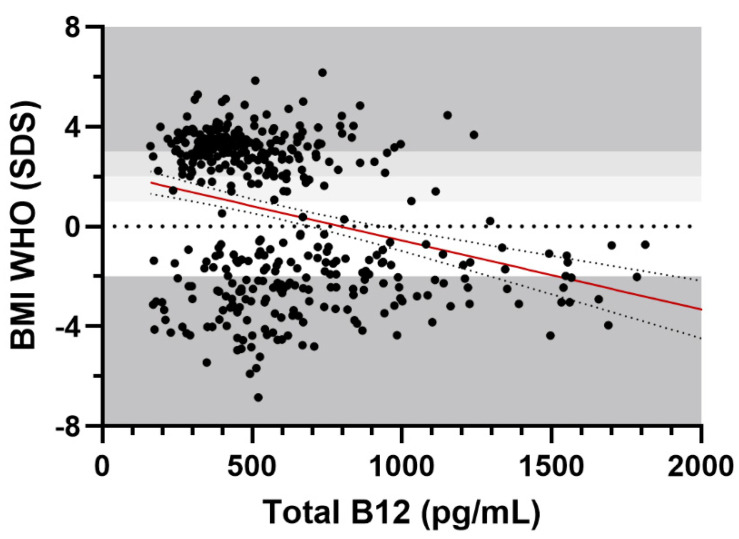
Scatter plot of total vitamin B12 levels according to BMI SDS. Note: weight groups’ borders are demarcated in shades of grey, to indicate underweight, normal weight, overweight, obesity and severe obesity on the y axis. The best-fit line is represented in red, alongside its 95% confidence bands.

BMI SDS was negatively correlated with serum cobalamin in the whole cohort (r = −0.32, *p* < 0.001, BCa 95% −0.38, −0.25), as shown in Figure 2. The association was unchanged after adjusting for age and sex in a partial correlation analysis (r = −0.31, *p* < 0.001, BCa 95% −0.38, −0.24).

**Figure 3:**
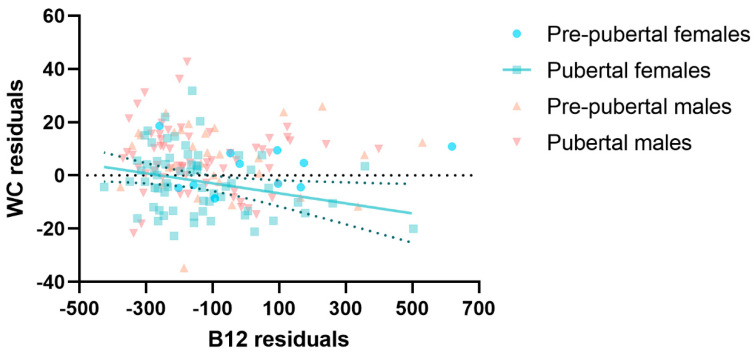
Scatter plot of the residuals of waist circumference according to total vitamin B12 levels, sex and pubertal status. Note: residuals represent the difference between the observed and the estimated values of the variables of interest, adjusting for age with linear regression models. The best-fit line is represented in green, alongside its 95% confidence bands.

No relationship was found between serum cobalamin levels and WC in the whole study cohort. However, after stratifying the patients according to sex and pubertal stage, a significant negative correlation between vitamin B12 and WC was evident in pubertal girls, adjusting for age in a partial correlation analysis (r = −0.33, *p* = 0.008, BCa 95% −0.53, −0.11), as shown in Figure 3.

### 2.2. Glucose Metabolism and Insulin Resistance According to Vitamin B12 Levels

Overall, an altered glucose metabolism was present in 14.9% of patients. Specifically, IFG was diagnosed in 4.3% (n = 26), IGT in 10.1% (n = 60) and T2DM in 0.5% (n = 2) of patients.

When exploring hepatic and whole-body IR using a linear mixed-effects model approach, with sex and pubertal status as fixed effects and BMI SDS as random effect, an inverse correlation between cobalamin levels and HOMA-i was observed in males (B = −0.003 (−0.007, −0.0001), *p* = 0.039) but not in females (B = 0.002 (−0.001, 0.005), *p* = 0.260). No significant correlation was present between cobalamin levels and the Matsuda index in the whole cohort as well as according to gender or pubertal status (*p* > 0.05).

### 2.3. Lipid Profile, Hepatic Steatosis and Liver Function Tests According to Vitamin B12 Levels

No significant correlation was found between serum B12 levels in the whole sample, after adjusting for age, sex and BMI SDS, with total (r = −0.03, *p* = 0.60), HDL (r = 0.02, *p* = 0.73) or LDL cholesterol (r = −0.03, *p* = 0.59) or serum triglycerides (r = −0.07, *p* = 0.21).

**Figure 4:**
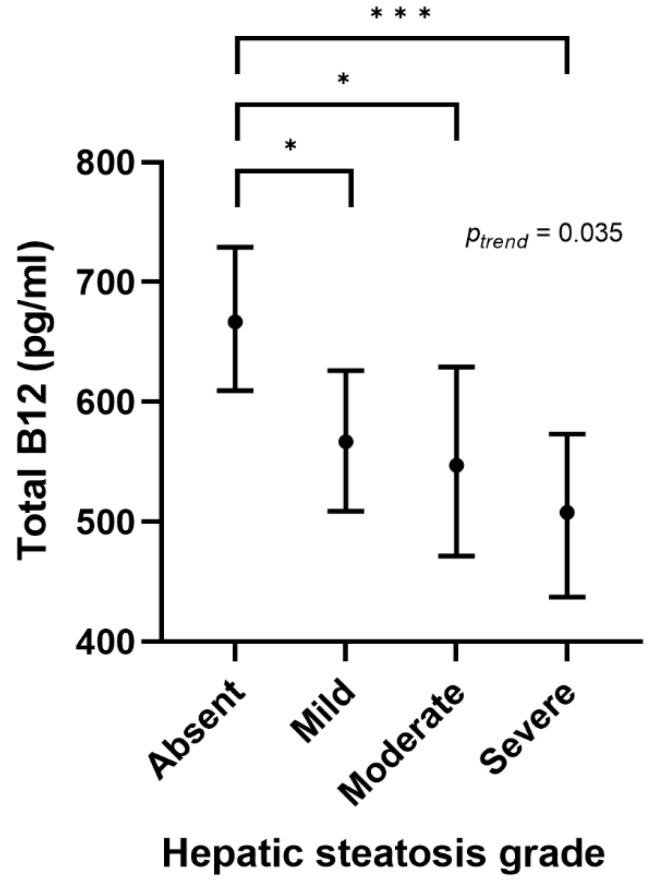
Serum total vitamin B12 levels according to ultrasound-derived hepatic steatosis grade. Note: data are adjusted for BMI SDS and represent the adjusted means and their respective BCa 95% CI. (* *p* ≤ 0.05 and *** *p* ≤ 0.001).

Hepatic steatosis, as assessed by liver ultrasound, was present in the 57.9% of study subjects. Specifically, it was mild in 29.8%, moderate in 16.5% and severe in 11.6% of cases and was more severe in males as compared to females (*p* < 0.001). When adjusting for BMI SDS, serum cobalamin levels tended to be lower in subjects with more severe hepatic steatosis (*p_trend_* = 0.035), as shown in Figure 4.

Higher hepatic IR and lower whole-body insulin sensitivity were observed with increasing hepatic steatosis severity as shown by HOMA-i and the Matsuda index, respectively. The estimated marginal means (95% CI) for HOMA-i according to hepatic steatosis group were as follows: absent, 3.62 (3.06, 4.70); mild, 4.17 (3.66, 4.70); moderate, 5.19 (4.49, 5.99); and severe, 6.98 (5.65, 8.38). A higher HOMA-i was present in the moderate steatosis group vs. absent (*p* = 0.0018) and in the severe steatosis group vs. absent (*p* < 0.001) and mild steatosis (*p* = 0.001). The estimated marginal means (95% CI) for the Matsuda index according to hepatic steatosis groups were as follows: absent, 4.40 (3.49, 5.48); mild, 3.95 (3.31, 4.66); moderate, 3.19 (2.60, 3.91); and severe, 2.50 (1.96, 3.13).

A lower Matsuda index was observed in the group with severe hepatic steatosis vs. those with absent (*p* = 0.017) and mild steatosis (*p* = 0.010).

As for liver function tests, both AST (r = 0.21, *p* < 0.001) and ALT (r = 0.22, *p* < 0.001) were significantly associated with total B12 levels in the population. However, when exploring the relationship according to weight group, B12 was significantly associated with AST and ALT only in the extreme groups, i.e., in underweight (r = 0.22 and 0.24, *p* = 0.002 and <0.001, respectively) and severe obesity groups (r = 0.24 and 0.32, *p* = 0.002 and <0.001). No association was found with GGT levels.

## 3. Discussion

Cobalamin is an essential, food-derived vitamin and has a fundamental role in several metabolic pathways necessary for cellular and tissue integrity. In the cytosol, cobalamin is processed to its catalytic forms: *5′ adenosyl-cobalamin* (AdoCbl) and *methyl-cobalamin* (MeCbl), involved in cell energy metabolism and multiple reactions of one-carbon metabolism, including nucleotide synthesis and DNA methylation.

Indeed, the intracellular fate of vitamin B12 is primarily associated with its role as a cofactor for two key enzymes: methionine synthase and methylmalonyl-CoA mutase. The former catalyses the conversion of homocysteine to methionine. This reaction is a key step in the methionine cycle, which is crucial for the synthesis of S-adenosylmethionine (SAM), a universal methyl donor involved in various cellular methylation reactions. Methionine is an essential amino acid that serves as the precursor for protein synthesis, and its production is vital for maintaining cellular functions. The latter enzyme is involved in the catabolism of certain amino acids and fatty acids. Methylmalonyl-CoA is converted to succinyl-CoA through the action of methylmalonyl-CoA mutase. This reaction is important for the metabolism of propionate, a component of some amino acids and odd-chain fatty acids. Methionine produced with the help of vitamin B12 is crucial for DNA synthesis. Adequate methionine levels contribute to the availability of S-adenosylmethionine (SAM), which is a methyl donor for DNA methylation reactions. DNA methylation plays a key role in regulating gene expression and maintaining genomic stability. Thus, vitamin B12 indirectly influences these processes through its involvement in methionine synthesis. Furthermore, vitamin B12 is essential for the maintenance of the nervous system. It is involved in the synthesis of myelin, the protective sheath around nerves. Deficiencies in vitamin B12 can lead to neurological symptoms, including peripheral neuropathy, cognitive impairment and, in severe cases, irreversible neurological damage [41].

Furthermore, vitamin B12 deficiency contributes significantly to the metabolic impairment of people with obesity.

The importance of vitamin B12 in regulating key human metabolic pathways has been confirmed by in vitro studies on human primary adipocytes and hepatocyte cell lines. Low cobalamin levels result in reduced *methionine* synthesis, reduced synthesis of the derived *S-adenosyl-methionine* (SAM) and increased production of *Hcy* and *S-adenosyl-homocysteine* (SHcy). The resulting reduction in the SAM/SHcy ratio is associated with hypomethylation and upregulation of promoter regions of genes involved in cholesterol biosynthesis and metabolism, such as *sterol regulatory element-binding proteins* (SREBF 1-2), *low-density lipoprotein receptor* (LDLR) and *3-hydroxy-3-methylglutaryl CoA* (HMG-CoA) *reductase* [14]. Cobalamin deficiency is also associated with increased intracellular triglycerides, through increased synthesis and uptake of fatty acids and decreased fatty acid β-oxidation [2,14,42]. Intracellular accumulation of MMA, due to low B12 levels, inhibits *carnitine palmitoyltransferase 1* (CPT1), a rate-limiting enzyme for fatty acid β-oxidation [2,43,44].

Conditions of malabsorption, such as bariatric surgery, atrophic autoimmune gastritis, small-intestinal bacterial overgrowth and inflammatory bowel diseases, can affect vitamin B12 levels, leading to a deficient state. Among commonly prescribed medications, metformin, proton pump inhibitors and histamine H2 receptor blockers are also associated with reduced absorption [45].

The present study aimed to explore the relationship between serum B12 levels and metabolic phenotype in a relatively large population of pre-pubertal and pubertal children, adolescents and young adults, spanning across the whole spectrum of body weight.

As expected, subjects in the underweight and normal weight groups presented higher B12 levels when compared to the groups with obesity and severe obesity. Furthermore, low levels of vitamin B12 were associated with a higher body weight, expressed as BMI SDS. This negative correlation was confirmed after adjusting for potential confounding by age and gender, which are known to affect B12 concentrations [46]. Findings of our study are in line with the available evidence in pre-clinical and clinical models, describing a higher adiposity and a worse body composition in offspring of B12-deficient mothers and reduced levels of plasma cobalamin in obese children, adults and pregnant women [16,19,20,21,22]. Nonetheless, when exploring the specific relationship between B12 and visceral adiposity, as represented by WC, no significant association was found in the whole cohort. However, subgroup analyses, taking into account gender and pubertal status, revealed a significant inverse relationship between B12 concentrations and WC in pubertal girls, after adjusting for age. This finding suggests a mediation effect of sex steroids, specifically oestrogens, in modulating the effects of B12 on body composition and visceral adiposity in particular [47].

Findings of our study confirm a frequency of impaired glucose metabolism (IFG, IGT and T2DM) in young white non-Hispanic individuals with obesity as high as 15% [48]. As to the relationship between vitamin B12 status and IR that characterises altered glucose metabolism in these individuals, we observed a negative association between hepatic IR and B12 levels, which was evident only in males, regardless of pubertal status. This finding can be explained by the differences arising from the sexual dimorphism of pituitary–gonadal and somatotopic axes on glucose metabolism in males and females [48,49]. In this regard, data from the literature are controversial. Studies in offspring born to B12-deficient mothers, PCOS women and in adolescents with obesity found a negative association between the two [23,50,51]. Lower circulating B12 was associated with higher prevalence of obesity and increased IR during pregnancy in a non-diabetic British population [52] and with overweight and obesity, but not with IR or metabolic syndrome, in another study on 976 Turkish individuals of various degrees of body weight [53]. Furthermore, in our population, a more severe hepatic fatty infiltration at ultrasound was observed in males, associated with higher hepatic IR and reduced whole-body sensitivity to the hormone. As such, in patients with hepatic steatosis, low levels of B12 may contribute to the worsening of fatty liver disease and to the onset and progression of altered glucose metabolism.

After adjusting for BMI SDS, patients with more severe hepatic steatosis at ultrasound showed significantly lower B12 levels. The hepatic metabolic impact of B12 status might be explained owing to cobalamin’s role in regulating lipolysis and lipogenesis, specifically in hepatocytes [7,14]. B12 deficiency can also alter one-carbon metabolism and cause mitochondrial dysfunction, both responsible for the development of hepatic fibrosis [9,31].

Despite the above-described effects on lipid metabolism, an association between low B12 levels and a worse serum lipid profile were not observed in our population, when assessing total, HDL and LDL cholesterol and triglycerides. This may be due to the intrinsically different nature of the study population, composed of subjects between the ages of 5 and 25, and to the higher degree of metabolic flexibility and resilience in this group of patients [54,55].

Hepatic dysfunction is a common complication in AN because of hepatocyte autophagy, oxidative stress, MASLD, hepatic hypoperfusion and glycogen depletion. In patients with AN, the relative overflow of nutrients to the liver during the refeeding phase may become an insult for parenchyma and result in hypertransaminasemia. The extent of the insult is inversely proportional to the severity of undernutrition [40]. The relationship between AST, ALT and cobalamin in the refeeding phase of anorexic patients has been explored in two studies [37,56] in which high B12 plasma levels were described as an earlier marker of liver damage with respect to hypertransaminasemia, suggesting that attention be paid to B12 levels in the refeeding of these patients. Indeed, our study confirms a strong positive correlation of cobalamin levels with increased serum AST and ALT in the extreme groups of the studied population (underweight and subjects with severe obesity). In this frame, either high or low levels of B12 may predict the risk of severe liver derangement up to end-stage liver disease.

The current study presents some strengths and limitations. It is the first study designed to study the association between total cobalamin levels and metabolic phenotype in a relatively large cohort of children, adolescents and young adults, spanning across the whole body-weight spectrum, from underweight to severe obesity. Furthermore, the single-centre design and the sample size allowed us to take into account and adjust for potential confounding factors, such as age, gender, BMI and pubertal status.

On the other hand, the cross-sectional nature prevents us from assessing the causality of the above-described associations. Some data (i.e., fasting plasma insulin and HbA1_C_) were unavailable in the underweight and normal weight groups, possibly limiting the study conclusions. It should also be acknowledged that WC measurement, although simple and widely available in clinical practice, is less accurate in estimating visceral adiposity when compared to other methodologies (e.g., dual-energy X-ray absorptiometry scanning) and is an operator-dependent methodology.

## 4. Materials and Methods

### 4.1. Study Participants

This study was performed at the academic paediatric hospital ”Bambino Gesú” in Rome, Italy. Outpatients were referred to the ”Nutritional educational therapy” section of the ”Endocrinology and Diabetology” and ”General Paediatrics” units, from October 2021 to July 2023. The inclusion criteria were as follows: age between 5 and 25 years; complete dataset including data on serum vitamin B12 levels, clinical, auxological, metabolic and liver ultrasounds. The exclusion criteria were as follows: (1) syndromic or monogenic obesity; (2) presence of other known genetic or chromosomal conditions; (3) chronic systemic diseases (such as cystic fibrosis, congenital heart disease, cerebral palsy, etc.); (4) history of bariatric surgery; (5) history of sellar or parasellar disease, surgery and radiotherapy; (6) malabsorption conditions (such as coeliac disease, Crohn’s disease, etc.); and (7) use of drugs inducing weight-loss drugs or those impacting on glucose metabolism (such as metformin, orlistat, liraglutide, etc.).

Initially, 784 patients were screened, of whom 183 were excluded due to various exclusion criteria. Thus, 601 subjects were enrolled. They were stratified according to BMI SDS into the following five groups: (1) underweight (n = 121, <−2 SDS), (2) normal weight (n = 75, −2 ≤ SDS < 1), (3) overweight (n = 44, ≥1 SDS < 2), (4) obese (n = 135, ≥2 SDS < 3) and (5) severely obese (n = 226, ≥3 SDS), according to WHO criteria [57]. Patients were further categorised according to gender (males and females) and pubertal status (prepubertal and pubertal, from Tanner stage II onward). All patients underwent a thorough clinical examination and metabolic evaluation. An endocrine disease affecting body weight or eating behaviour was excluded as part of routine clinical care.

### 4.2. Metabolic Evaluation

Blood samples were obtained in the early morning (08:00–09:30 h) by venipuncture, after an overnight fast, and immediately centrifuged and assayed. Red blood cell, white blood cell and platelet counts were determined and haemoglobin was quantified in all patients. Serum cobalamin levels were determined by electro chemiluminescent assays (ECLIA). Both glucose and insulin were measured under basal conditions and after 30, 60, 90 and 120 min as part of an oral glucose tolerance test (OGTT) with 1.75 g/kg (up to a maximum of 75 g) of glucose. OGTT measures allowed the evaluation of glucose metabolism status (normal tolerance, impaired fasting glucose (IFG), impaired glucose tolerance (IGT) and type 2 diabetes mellitus (T2DM)), according to the American Diabetes Association 2023 standards of care [58]. Glycated haemoglobin was assessed by high-performance liquid chromatography (HPLC). Total, HDL and LDL cholesterol and triglyceride levels were measured by enzymatic colorimetric assays. Glucose was measured by the hexokinase–G6PD method, whereas insulin was determined by ECLIA. Transaminases (aspartate aminotransferase, AST and alanine aminotransferase, ALT) and γ-GT were measured by the International Federation of Clinical Chemistry and Laboratory Medicine method at 37 °C. The Homeostatic Model Assessment index (HOMA-i) of insulin resistance (IR) was calculated as a hepatic IR marker [59] and the Matsuda index was derived from the OGTT values as a marker of whole-body insulin sensitivity [60].

### 4.3. Clinical and Ultrasonographic Evaluations

Anthropometrics was evaluated using a Harpenden stadiometer to measure height, calibrated scales to determine weight and a body measuring tape for waist circumference (WC). For each patient, the BMI was calculated. Height, weight and BMI values were transformed into standard deviation scores (SDS) according to WHO growth reference parameters [57], using the free software Growth4 (http://www.weboriented.it/gh4/ (accessed on 1 September 2023)) provided by the Italian Society for Paediatric Endocrinology and Diabetology. Body surface area (BSA) was calculated according to Haycock’s formula for all age groups [61]. Qualitative ultrasonographic examination of hepatic steatosis was performed by a single operator using a Siemens Acuson X700 device coupled with a 4C1 convex transducer (1.0–4.0 MHz). Steatosis was categorised as absent or present and graded as mild, moderate or severe through B-mode ultrasound findings [62].

### 4.4. Statistical Analysis

Data are expressed as means and/or medians, as appropriate, and as standard deviations (SD), 95% confidence intervals (CIs) and 25–75% interquartile ranges (IQR). Data distributions were visually inspected by analysing the respective histograms and normality plots. Data were tested with Brown–Forsythe and Welch ANOVA tests for unequal variances, corrected for multiple comparisons (Dunnett T3), with Kruskal–Wallis H tests for non-normal data, and with linear regressions, partial correlations and linear mixed-effects models, to assess trends and associations between vitamin B12 levels and parameters of interest. Models were adjusted for age, gender and pubertal status as appropriate, after bootstrapping on 2000 samples. The level of statistical significance is set to 0.05 and *p*-values for two-tailed tests are reported, alongside Wald 95% or bias-corrected and accelerated (BCa) CIs. Data are visually represented with box–whisker plots as the median (black lines), 25–75% IQR (boxes) and 2.5–97.5th percentiles (whiskers) and with scatter plots, representing the best-fit line and its 95% confidence bands for significant linear regressions.

All statistical computations were conducted with IBM SPSS Statistics for Windows (version 28, IBM Corp. Chicago, IL, USA) and GraphPad Prism for Windows (version 8.3.0, GraphPad Software, LLC, Boston, MA, USA).

Written informed consent was obtained from all the participants of legal age able to do so whereas, in minors, it was obtained from the participants’ parents. The study was approved by the Ethics Committee of Paediatric Children Hospital “Bambino Gesù” IRCCS (Ref. 2050_OPBG_2020) on the 23 March 2022.

## 5. Conclusions

In conclusion, the present study explores the association between serum vitamin B12 levels and metabolic status in a large paediatric population, revealing how low cobalamin levels are associated with higher body weight and visceral adiposity and worse IR and hepatic steatosis and may serve as marker of liver injury. Future studies are needed to confirm the association of cobalamin with obesity and metabolic impairment of patients across the BMI spectrum, also taking into account Hcy, MMA and holo-TC levels, to better investigate B12 status [63]. Furthermore, intervention studies will be needed to assess causality and unravel the potential benefits of vitamin B12 supplementation on the metabolic phenotype of obese and severely obese young patients.

## Figures and Tables

**Table 1:** General, anthropometric and biochemical data according to weight group.

	Underweight	Normal Weight	Overweight	Obese	Severely Obese	*p*
N (%)	121 (20.1)	75 (12.5)	44 (7.3)	135 (22.5)	226 (37.6)	0.059
Age, year	14.9 ± 2.1	13.9 ± 2.3	14.0 ± 2.3	13.2 ± 2.7	11.7 ± 3.7	0.102
Males, %	6.6	12.0	45.5	45.9	61.1	**<0.001**
Height, cm *	159.0 [153.0, 164.0]	156.0 [151.0, 164.0]	156.7 [149.3, 163.0]	158.5 [147.6, 165.9]	155.0 [140.3, 164.3]	**0.005**
Weight, kg	35.2 ± 5.8	41.2 ± 7.2	60.5 ± 11.8	72.4 ± 18.9	82.8 ± 30.4	**<0.001**
Body mass index, kg/m^2^ *	14.1 [13.0, 15,0]	16.7 [15.8, 17.6]	24.5 [22.7, 26.5]	28.9 [25.7, 31.9]	35.0 [30.3, 39.2]	**<0.001**
Body mass index, SDS	−3.3 ± 1.0	−1.2 ± 0.6	1.6 ± 0.3	2.6 ± 0.3	3.6 ± 0.6	**<0.001**
Body surface area, m^2^	1.2 ± 0.1	1.3 ± 0.2	1.6 ± 0.2	1.8 ± 0.3	1.8 ± 0.4	**0.007**
SBP, mmHg *	101.0 [94.0, 108.0]	102.0 [95.8, 112.0]	118.5 [110.0, 120.0]	120.0 [112.5, 124.0]	120.0 [115.0, 128.0]	**<0.001**
DBP, mmHg	65.2 ± 8.7	64.8 ± 9.4	68.3 ± 7.1	68.5 ± 8.7	71.5 ± 8.7	0.184
**Biochemical evaluation**						
FPG, mg/dL	78.2 ± 11.4	80.0 ± 14.4	82.2 ± 9.0	86.7 ± 13.3	87.0 ± 8.9	0.466
FPI, µUI/mL *	/	/	12.0 [9.3, 17.0]	17.3 [10.0, 26.2]	23.1 [13.3, 31.9]	**0.015**
HOMA-i	/	/	2.43 [1.91, 3.61]	3.50 [2.26, 5.39]	4.94 [2.69, 7.22]	**<0.001**
Matsuda index	/	/	3.27 [2.16, 6.61]	2.74 [1.85, 4.43]	2.42 [1.66, 3.93]	**<0.001**
HbA1_C_, mmol/mol	/	/	32.9 ± 2.8	33.8 ± 4.4	34.8 ± 4.5	0.391
Total cholesterol, mg/dL *	163.0 [135.0, 200.0]	151.5 [125.8, 185.3]	153.5 [137.0, 167.8]	157.0 [133.0, 175.0]	154.0 [134.0, 173.0]	**<0.001**
HDL cholesterol, mg/dL *	62.0 [51.0, 75.0]	57.0 [48.0, 68.0]	51.0 [43.8, 59.5]	46.5 [41.3, 55.0]	45.0 [39.8, 52.0]	**<0.001**
LDL cholesterol, mg/dL	108.8 ± 51.9	86.4 ± 32.6	91.9 ± 24.3	96.1 ± 25.4	94.9 ± 26.2	**0.027**
Triglycerides, mg/dL *	72.0 [55.5, 92.3]	70.5 [58.3, 85.8]	65.0 [53.3, 80.5]	79.0 [59.0, 112.0]	87.0 [66.0, 123.0]	**<0.001**
AST, U/L *	21.0 [18.0, 27.0]	19.0 [16.0, 25.0]	18.0 [16.0, 23.0]	22.0 [18.0, 27.0]	24.0 [19.0, 31.0]	**<0.001**
ALT, U/L *	16.0 [11.5, 26.5]	15.0 [11.0, 19.0]	14.0 [11.0, 17.0]	19.0 [15.0, 28.0]	23.0 [17.0, 35.5]	**<0.001**
GGT, U/L *	11.0 [8.0, 15.0]	9.0 [6.0, 11.0]	12.5 [10.0, 15.8]	13.0 [10.0, 18.0]	17.0 [13.0, 22.0]	**<0.001**
Vitamin B12, pg/mL *	596.0 [450.3, 871.8]	718.0 [522.0, 933.0]	609.5 [430.5, 682.0]	446.0 [328.3, 557.8]	417.0 [349.3, 565.8]	**<0.001**

*Note:* data are presented as means ± SDs and have been tested via ANOVA; however, parameters marked by * show a non-normal distribution, are presented as the median [25–75% IQR] and have been tested via the Kruskal–Wallis H test. Significant *p*-values are represented in bold.

## Data Availability

The data presented in this study are available upon reasonable request from the corresponding author.

## Abbreviations

ALT	alanine aminotransferase
AN	anorexia nervosa
AST	aspartate aminotransferase
BCa	bias-corrected and accelerated
BMI	body mass index
BSA	body surface area
CIs	confidence interval(s)
DBP	diastolic blood pressure
DXA	dual-energy X-ray absorptiometry
ECLIA	electrochemiluminescence assay
FPG	fasting plasma glucose
FPI	fasting plasma insulin
γ-GT	gamma-glutamyltransferase
Hcy	homocysteine
Holo-TC	holo-transcobalamin
HOMA-i	homeostasis model assessment insulin resistance index
HPLC	high-performance liquid chromatography
IF	intrinsic factor
IFG	impaired fasting glucose
IGT	impaired glucose tolerance
IQR	interquartile ranges
IR	insulin resistance
MAFLD	metabolic-associated fatty liver disease
MASH	metabolic-associated steatohepatitis
MMA	methylmalonic acid
OGTT	oral glucose tolerance test
SBP	systolic blood pressure
SD	standard deviation
SDS	standard deviation scores
T2DM	type 2 diabetes mellitus
TC	transcobalamin
WC	waist circumference
WHO	World Health Organization
